# Obligation or Desire: Variation in Motivation for Compliance With COVID-19 Public Health Guidance

**DOI:** 10.3389/fpsyg.2021.647830

**Published:** 2021-07-06

**Authors:** Ting Ai, Glenn Adams, Xian Zhao

**Affiliations:** ^1^Department of Psychology, University of Kansas, Lawrence, KS, United States; ^2^Rotman School of Management, University of Toronto, Toronto, ON, Canada

**Keywords:** COVID-19, prosocial motivation, culture, obligation, compliance

## Abstract

Why do people comply with coronavirus disease 2019 (COVID-19) public health guidance? This study considers cultural-psychological foundations of variation in beliefs about motivations for such compliance. Specifically, we focused on beliefs about two sources of prosocial motivation: desire to protect others and obligation to society. Across two studies, we observed that the relative emphasis on the desire to protect others (vs. the obligation to the community) as an explanation for compliance was greater in the United States settings associated with cultural ecologies of abstracted independence than in Chinese settings associated with cultural ecologies of embedded interdependence. We observed these patterns for explanations of psychological experience of both others (Study 1) and self (Study 2), and for compliance with mandates for both social distancing and face masks (Study 2). Discussion of results considers both practical implications for motivating compliance with public health guidance and theoretical implications for denaturalizing prevailing accounts of prosocial motivation.

## Introduction

The coronavirus disease 2019 (COVID-19) pandemic has posed an enormous threat to both individuals and societies all over the globe. In response, dozens of countries have declared strict measures (e.g., self-isolation, travel restrictions, social distancing, and wearing face masks) to curb the spread of the new coronavirus. The stakes are high. Until most people in a community have received the vaccine, the key to “flatten the curve” is for individuals to comply with these measures (Luttrell and Petty, 2020; Pfattheicher et al., 2020). Evidence suggests that outbreaks were relatively more severe in settings where communities failed to enact, enforce, or comply with mandates for face masks, social distancing, restrictions on movement, or other public health guidance (Cheng et al., 2020). Some research suggests that cultural–psychological features associated with modern individualist ecologies (e.g., opposition to the hierarchy, Atalay and Solmazer, 2021; or relational mobility, Salvador et al., 2020) may afford such failures to enact or comply with public health mandates, resulting in more negative health outcomes related to COVID-19 (Güss and Tuason, 2021; Webster et al., 2021).

Why do people comply with COVID-19 public health mandates? Some motivations for compliance with COVID-19 measures are self-serving. For example, people comply to protect themselves from illness associated with the coronavirus, to avoid punishment for violating mandatory measures, or to obey authorities (Murphy et al., 2020). The focus of the current studies is another category of motivations for compliance with COVID-19 measures, prosocial motivation. Indeed, researchers have shown that activation of prosocial motivation can be as effective or more effective than activation of self-serving motivation for promoting compliance with social distancing and wearing face masks (Jordan et al., 2020; Luttrell and Petty, 2020; Pfattheicher et al., 2020; Miyajima and Murakami, 2021). In this study, we consider whether understandings of prosocial motivation, as a desire to help others or obligation to the community, vary in theory-relevant ways across settings.

Standard accounts of prosocial motivation often define it as “the desire to benefit other people” (Grant, 2008). Drawing upon self-determination theory (SDT) (Deci and Ryan, 2000), standard accounts typically emphasize the importance of autonomy in prosocial motivation and behaviors (e.g., Grant, 2008; Weinstein and Ryan, 2010). For example, Grant (2008) distinguished between autonomous/intrinsic and non-autonomous/controlled prosocial motivation. The former refers to an authentic and personal desire to benefit others that emanates from the intrinsic self and is congruent with one's value. In contrast, the latter has its basis in coercion from social pressure and feelings of obligation. Grant (2008) further argued that autonomous prosocial motivation is more likely to produce persistence, performance, and productivity, because it enables individuals to fulfill their basic psychological needs for autonomy, competence, and relatedness. Similarly, Weinstein and Ryan (2010) showed that autonomous motivation for prosocial behavior predicted higher well-being for both the helpers and recipients, compared with controlled motivation.

Standard accounts tend to portray observed patterns of prosocial motivation or other aspects of psychological experience as the just-natural outgrowth of inborn tendencies. In contrast, a cultural psychological account considers the cultural–ecological affordances that provide the foundation for observed patterns. In terms of the topic of the current studies, a cultural psychological perspective proposes that the standard approach to prosocial motivation as personal desire may reflect particular affordances of the Western, Educated, Industrialized, Rich, and Democratic (e.g., WEIRD; Henrich et al., 2010) settings that disproportionately constitute the base of hegemonic psychological science. The affordances of WEIRD settings may obscure equally valid or important constructions of prosocial motivation, such as social obligation, that are underrepresented in mainstream psychological studies.

A useful way of understanding cultural–ecological variation in psychological experience concerns a distinction between independence or abstraction from context and interdependence or embeddedness in context (Adams and Kurtiş, 2018). Cultural ecologies of abstracted independence, which are prominent in WEIRD settings of Eurocentric modernity, afford an ontological experience as an autonomous and bounded entity, inherently separate from social and physical context, composed of internal attributes, such as personality, abilities, and preferences (Markus and Kitayama, 1991; Markus et al., 1997; Adams and Kurtiş, 2018). These cultural ecologies afford an experience of interpersonal connection as a voluntary creation of inherently separate, self-contained entities. Given a lack of inherent connection, people feel a sense of freedom not only to initiate and to maintain connections they find pleasurable, but also to dissolve connections that they no longer find satisfying (Adams and Kurtiş, 2018). In this view, role-related expectations, obligation, duties, or other social influence that impinge on a person from social formations, such as the broader community, that a person has not voluntarily constructed are likely to hinder a sense of individual agency and control and thereby dampen motivation (Miller et al., 2011). The ideal of independence is in line with the key claim within SDT that actions or choices that follow from self-determined or less controlled motivations are associated with higher positive affect (Baard et al., 2004; La Guardia and Patrick, 2008).

In contrast, cultural ecologies of embeddedness and interdependence, which are prominent in many non-WEIRD settings, afford an ontological experience as a fluid node of interpersonal connection inherently embedded within the social and physical context (Markus and Kitayama, 1991; Markus et al., 1997). These cultural ecologies afford an experience of interpersonal connection as the default condition of human existence (Adams, 2005). Rather than a sense of freedom to create and dissolve relationships as a function of personal satisfaction, people must manage existing connections to maintain communal harmony and social order. In this view, the experience of obligation is an inevitable fact of social life and even something compatible with personal inclinations rather than an onerous burden (Vasudev and Hummel, 1987; Miller, 1994; Buchtel et al., 2018; Goyal et al., 2019; Esiaka et al., 2020). The implication is that in cultural worlds of embedded interdependence, people may experience similar or greater motivation for action that follows from the social obligation to the broader community as for action that follows from a personal desire to help particular others.

A cultural psychology approach highlights cultural-ecological variation in the experience of prosocial motivation. Cultural ecologies of abstracted independence afford an experience of empathy and concern for particular others as the source of motivation for intentions to help. They afford an experience of obligation to the community as something at odds with relatively autonomous, freely chosen action and, therefore, as a less effective source of prosocial motivation. In contrast, cultural ecologies of embedded interdependence afford an experience of obligation as a natural way of social life, in which obligation to the community is an authentic source of prosocial motivation that people find as compelling as the desire to protect particular others.

Support for these theoretical propositions comes from previous research on cultural variation in understandings of prosocial motivation (e.g., Miller, 1994; Miller et al., 2011; Buchtel et al., 2018; Gherghel et al., 2020). For example, Miller et al. (2011) found that expectations to help family and friends were positively correlated with satisfaction and choice only among the Indian participants and not among the United States participants. Similarly, Buchtel et al. (2018) found that the perception of greater warmth, competence, sense of choice, and enjoyment in desire-motivated helpers compared with obligation-motivated helpers was more pronounced among participants from WEIRD settings than among participants from East Asian settings.

Indirect support also comes from research that examines cultural variation in the effectiveness of health messages that administrators frame as promoting gains vs. preventing losses (e.g., Lee et al., 2000; Uskul et al., 2009). This line of research links cultural-ecological affordances for an ontological experience of abstracted independence to a relative emphasis on promotion-oriented motivations to express personal preferences and authentic desires. In contrast, this line of research links cultural-ecological affordances for an ontological experience of embedded interdependence to a relative emphasis on prevention-oriented motivations to meet social expectations (see also Higgins, 1996; Lee et al., 2000; Uskul et al., 2009). Public health appeals tend to be more persuasive if they are congruent with these motivational emphases. For example, researchers found that East Asian participants were more persuaded by prevention-focused or loss-framed health messages, whereas White British participants were more persuaded by promotion-focused or gain-framed health messages (Uskul et al., 2009).

Whereas the previous study has demonstrated cultural variation in beliefs about the consequences of obligation-motivated and desire-motivated prosocial behaviors, the current studies focus on cultural variation in beliefs about the motivational sources of prosocial behavior in the context of compliance with COVID guidance. Specifically, we considered the hypothesis that cultural ecologies of abstracted independence afford a distinction between types of prosocial motivation, such that desire to protect others figures more prominently than an obligation to the community in beliefs about compliance with COVID guidance. In contrast, because cultural ecologies of embedded interdependence promote less emphasis on the importance of choice in the experience of motivation and relationship (Adams et al., 2004; Savani et al., 2010), people are relatively unlikely to emphasize the desire to protect others over the obligation to the community (Miller et al., 2011). To test this hypothesis, we conducted two studies comparing responses of participants in United States and Chinese settings, which researchers have associated, respectively, with cultural ecologies of abstracted independence and embedded interdependence (e.g., Chua et al., 2005; Lalwani et al., 2009; Li et al., 2015).

## Study 1

The purpose of Study 1 was to investigate the relative weight of desire to protect others vs. the obligation to the community as explanations for compliance with COVID-19 public health guidance among Chinese and United States participants. We asked the participants to report beliefs about motivations of other people for compliance rather than their own motivations partly to reduce pressures for social desirability and self-presentation in self-report of own motivations (Everett et al., 2020) and because cultural beliefs are more evident in explanations of the psychological experience of others than explanations of one's own experience (Finch, 1987). We tested a primary hypothesis that the “standard” tendency to explain compliance as the result of personal desire to protect others rather than the social obligation to the community would be greater among United States participants than among Chinese participants.^1^

### Method

#### Participants

We conducted Study 1 at the end of April 2020. To determine sample size, we performed an a priori power analysis using the G^*^Power 3 computer program (Faul et al., 2007), which indicated that a sample of 200 participants would provide 80% power to detect a small effect (f = 0.1) or larger for the interaction between country and motivation type. We recruited 106 participants (61% women; age 18 to 86 with median of 56 years) residing in the United States from TurkPrime (https://www.cloudresearch.com) and 120 participants (64% women, age 18–57 with median of 30 years) residing in China from Sojump (https://www.wjx.cn), a Chinese crowdsourcing platform. Responses of the American participants to an open-ended item indicated that 74.5% self-identified as White, 15.1% Asian or Pacific Islander, 3.8% multi-ethnic, 2.8% Latino, 2.8% Black, and 0.9% American Indian/Alaskan Native. Responses of the Chinese participants indicated that 99.2% identified as Han.

#### Measures and Procedure

The participants reported their belief about the motivations of others for compliance with COVID-19 public health guidance *via* three measures. The first measure (*relative importance*) used a procedure adapted from previous research (Pronin and Kugler, 2010) that directed the participants to explain the relative importance of three motivations for compliance. One motivation option was *desire for self-protection*, which we defined as “People want to protect themselves from COVID-19 disease.” Another motivation option was *desire to protect others*, which we defined as “People feel empathy or care for others and desire to protect them from the disease.” A third motivation option was *obligation to the community*, which we defined as “As a member of the community, people feel a social responsibility to do their part in the community effort to stop the disease.” The participants allocated 100 percentage points to indicate the relative importance of each motivation, such that the sum of points across the three options was 100.

The second measure (*importance rating*) directed the participants to rate the importance of five reasons for compliance with COVID-19 guidance: *concern for punishment, obedience to authority, obligation to the community, desire to protect others*, and *desire for self-protection*. The participants used a scale from 1 (*not at all*) to 6 (*a great deal*) to make separate ratings of the extent to which each reason explains compliance of other people with COVID-19 public health guidance.

The third measure (*motivation to help*) assessed belief about motivations of others for wearing face masks *via* the Motivation to Help Scale (MHS) (Weinstein and Ryan, 2010), an 11-item scale that taps autonomous and controlled motivations for acts of helping. The participants first read a short vignette about a fictional person with a gender-neutral name who had been complying with guidance about wearing face masks to protect others. They then used a scale from 1 (*not at all*) to 6 (*a great deal*) to indicate their beliefs about the motivation of the person for compliance with reference to items from the MHS. Five items assessed autonomous motivation (e.g., “because s/he liked acting this way”; α = 0.72), and the remaining six items assessed controlled motivation (e.g., “because s/he felt he had to”; α = 0.72).

Finally, the participants reported whether they were living in an area with mandates for “social distancing” and “wearing a face mask.” They then completed demographic questions. We developed all materials in English, translated them into Chinese, and then back-translated into English to identify and correct any discrepancy.

### Results

Correlations among different motivations in three measures for the United States and Chinese participants appear in Tables 1, 2, respectively. To confirm variation in the relative weight of different motivations among settings, we conducted mixed-model ANOVAs with motivation type (for which the number of levels varied according to the measure) as the within-participant variable and country (China and United States) as the between-subject variable. To more precisely test the primary hypothesis regarding differences in the relative weight of motivations between the Chinese and United States participants, we conducted mixed-model ANOVAs with motivation type (*desire to protect others* or *obligation to the community*) as the two-level within-participant variable and country as the between-subject variable.

**Table 1:** Correlations among different motivations in three measures—United States participants.

**Variable**	**1**	**2**	**3**	**4**	**5**	**6**	**7**	**8**	**9**
**Relative importance**									
1. Desire									
2. Obligation	0.209^*^								
3. Self-protection	−0.823^**^	−0.728^**^							
**Importance rating**									
4. Desire	0.126	0.113	−0.154						
5. Obligation	0.002	0.344^**^	−0.204^*^	0.656^**^					
6. Self-protection	−0.327^**^	−0.189	0.339^**^	0.498^**^	0.348^**^				
7. Punishment	0.010	0.091	−0.060	0.008	0.265^**^	−0.020			
8. Authority	−0.003	0.239^*^	−0.137	0.195^*^	0.509^**^	0.121	0.525^**^		
**Motivation to help**									
9. Autonomous	0.065	0.077	−0.090	0.464^**^	0.491^**^	0.338^**^	0.157	0.314^**^	
10. Controlled	−0.083	0.141	−0.023	0.205^**^	0.314^**^	0.247^*^	0.285^**^	0.333^**^	0.488^**^

*Numbers indicate bivariate Pearson Correlation*.

* *p < 0.05*,

** *p < 0.01*.

**Table 2:** Correlations among different motivations in three measures—Chinese participants.

**Variable**	**1**	**2**	**3**	**4**	**5**	**6**	**7**	**8**	**9**
**Relative importance**									
1. Desire									
2. Obligation	0.059								
3. Self-protection	−0.710^**^	−0.745^**^							
**Importance rating**									
4. Desire	0.443^**^	0.305^**^	−0.511^**^						
5. Obligation	0.204^*^	0.447^**^	−0.452^*^	0.504^**^					
6. Self-protection	−0.123	−0.188^*^	0.215^*^	−0.037	0.117				
7. Punishment	−0.101	−0.145	0.170	−0.095	−0.152	−0.123			
8. Authority	−0.117	−0.151	0.184^*^	−0.013	−0.091	0.116	0.373^**^		
**Motivation to help**									
9. Autonomous	0.207^*^	0.212^*^	−0.288^**^	0.478^**^	0.429^**^	0.147	0.016	0.136	
10. Controlled	−0.041	−0.071	0.078	0.148	0.161	0.297^**^	0.188^*^	0.107	0.372^**^

*Numbers indicate bivariate Pearson Correlation*.

* *p < 0.05*,

** *p < 0.01*.

#### Relative Importance

**Figure 1:**
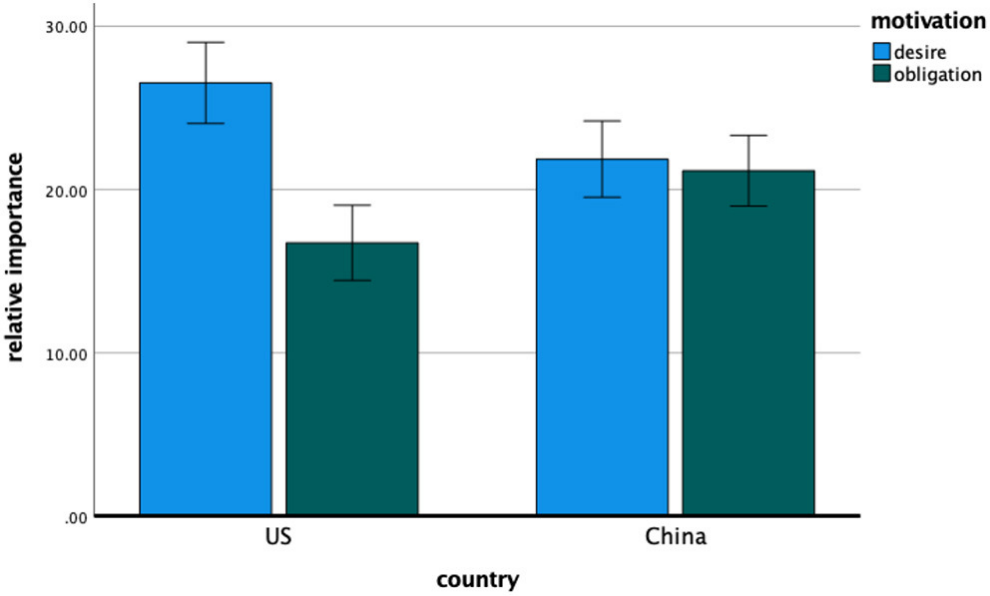
Study 1—the relative importance of desire and obligation motivation for American and Chinese participants. Error bars denote 95% confidence intervals of the mean.

Means and standard deviations for the relative importance measure appear in Table 3. The overall 2 (China, United States) × 3 (desire, obligation, self-protection) ANOVA revealed the anticipated interaction, *F*_(2, 448)_ = 3.47, *p* = 0.046, η^2^ = 0.015, indicating that the participants in Chinese and United States settings diverged in their relative emphasis on different motivations as an explanation for compliance with COVID-19 public health guidelines. More importantly, the focused analysis revealed a significant main effect of motivation type, *F*_(1, 224)_ = 22.95, *p* < 0.001, η^2^ = 0.093—such that the participants reported greater relative importance of *desire to protect others* (M = 24.06, SD = 13.16) than *obligation to the community* (M = 19.09, SD = 12.22)—qualified by the hypothesized interaction with country, *F*_(1, 224)_ = 17.17, *p* < 0.001, η^2^ = 0.071. Consistent with the primary hypothesis (and as shown in Figure 1), the relative emphasis on *desire to protect others* over *obligation to the community* as a motivation for compliance with COVID-19 guidance was greater for the United States participants, *t*(105) = 5.96, *p* < 0.001, Cohen's *d*_*z*_ = 0.58, 95% CI [0.37, 0.78], than for the Chinese participants, *t*(119) = 0.48, *p* = 0.629, Cohen's *d*_*z*_ = 0.04, 95% CI [−0.14, 0.22].

**Table 3:** Descriptive data for motivations included in relative importance measure among Chinese and American participants.

**Motivation**	**US**		**China**		***t* (224)**	**Cohen's *d_***s***_***
	**M (%)**	**SD**	**M (%)**	**SD**		
Desire	26.53	14.60	21.87	11.37	2.70^**^	0.360
Obligation	16.74	12.11	21.16	11.99	−2.75^**^	−0.366
Self-protection	56.71	20.82	56.97	17.01	−0.10	−0.014

** *p < 0.01*.

#### Importance Ratings

**Figure 2:**
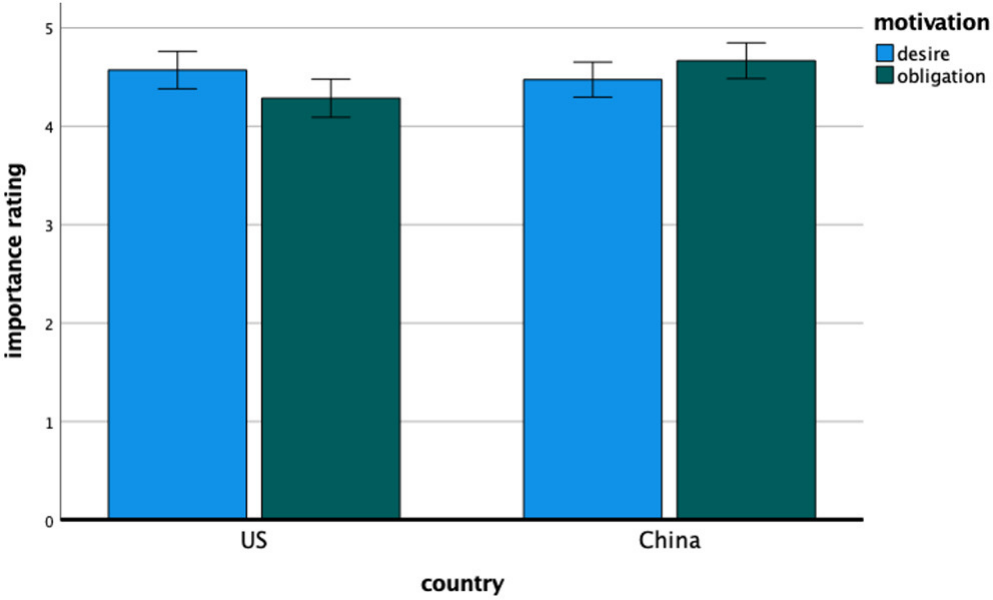
Study 1—the importance ratings of desire and obligation motivation for American and Chinese participants. Error bars denote 95% confidence intervals of the mean.

Means and standard deviations for the importance rating measure appear in Table 4. The overall 2 (China, United States.) × 5 (desire, obligation, self-protection, punishment, authority) ANOVA revealed the anticipated interaction, *F*_(4, 892)_ = 4.65, *p* = 0.004, η^2^ = 0.02, indicating that the participants in Chinese and United States settings diverged in their relative emphasis on different motivations as an explanation for compliance with COVID-19 guidelines. More importantly, the focused analysis again revealed the hypothesized interaction between motivation type and country, *F*_(1, 224)_ = 15.47, *p* < 0.001, η^2^ = 0.065. Consistent with the primary hypothesis (and as shown in Figure 2), the relative emphasis on *desire to protect others* over *obligation to the community* as a motivation for compliance with COVID-19 guidance was greater for the United States participants, *t*(104) = 3.19, *p* = 0.002, Cohen's *d*_*z*_ = 0.31, 95% CI [0.11, 0.51] than it was for the Chinese participants, who instead showed a tendency to emphasize obligation to the community over the desire to protect others, *t*(119) = −2.33, *p* = 0.021, Cohen's *d*_*z*_ = −0.21, 95% CI [−0.39, −0.03]. The overall country difference and main effect of motivation type were not significant.

**Table 4:** Descriptive data for motivations included in importance rating measure among Chinese and American participants.

**Motivation**	**US**		**China**		***t* (224)**	**Cohen's *d_***s***_***
	**M (%)**	**SD**	**M (%)**	**SD**		
Desire	4.58	1.10	4.48	0.92	0.83	0.110
Obligation	4.29	1.13	4.67	0.89	−2.82^**^	−0.377
Self-protection	5.43	0.94	5.72	0.70	−2.68^**^	−0.357
Punishment	2.69	1.50	3.41	1.28	−3.89^***^	−0.518
Authority	3.85	1.33	4.12	1.13	−1.64	−0.218

** *p < 0.01*,

*** *p < 0.001*.

#### Motivation to Help Scale

**Figure 3:**
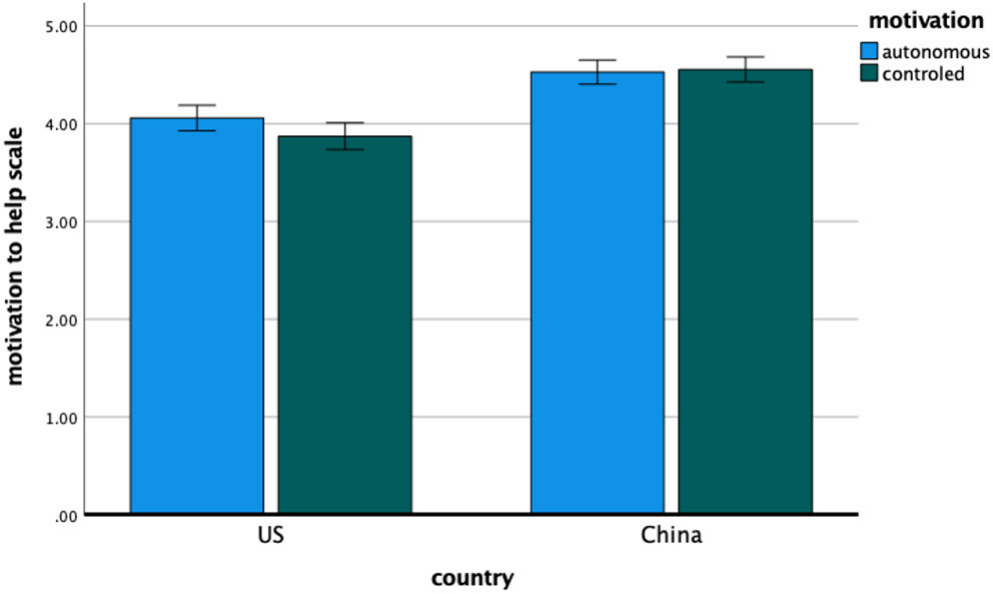
Study 1—the autonomous and controlled motivation from Motivation to Help Scale for American and Chinese participants. Error bars denote 95% confidence intervals of the mean.

Means and standard deviations for the MHS appear in Table 5. The analysis of MHS scores revealed a significant country difference, *F*_(1, 224)_ = 53.27, *p* < 0.001, η^2^ = 0.192, indicating that the Chinese participants generally reported higher motivations for compliance with COVID-19 measures. Yet again, we found the hypothesized interaction between motivation type and country, *F*_(1, 224)_ = 4.7, *p* < 0.05, η^2^ = 0.021. Consistent with the primary hypothesis (and as shown in Figure 3), the relative emphasis on desire over obligation as a motivation for compliance with COVID-19 guidance was greater for the United States participants, *t*(105) = 2.4, *p* = 0.018, Cohen's *d*_*z*_ = 0.23, 95% CI [0.04, 0.43] than for the Chinese participants, *t*(119) = −0.44, *p* = 0.663, Cohen's *d*_*z*_ = −0.04, 95% CI [−0.22,0.14]. The main effect of motivation type was not significant.

**Table 5:** Descriptive data for motivations included in Motivation to Help Scale among Chinese and American participants.

**Motivation**	**US**		**China**		***t* (224)**	**Cohen's *d_***s***_***
	**M (%)**	**SD**	**M (%)**	**SD**		
Autonomous	4.06	0.81	4.53	0.55	−5.16^***^	−0.688
Controlled	3.87	0.77	4.56	0.66	−7.18^***^	−0.957

*** *p < 0.001*.

#### Control Variables

The participants in the different countries reported differential emphasis on public health mandates. Whereas, the percentage of the United States participants who reported that they lived in an area with COVID-19 public health mandates was 97 % for “social distancing” and 65% for face masks, corresponding percentages for the Chinese participants were 50% and 97.5%. We included these variables along with gender, age, yearly income, and subjective socioeconomic status as covariates and re-ran the analyses described above. Results of these analyses do not qualify conclusions that we reported above for importance ratings and the measure of relative importance. In particular, the *p*-value associated with the focal interaction remained statistically significant (i.e., *p* < 0.05). This provides some assurance that observed results were not a function of demographic differences in the samples. In contrast, the focal interaction in the analysis of the motivation to help scale was not significant (*p* = 0.627).

#### Summary

Results of Study 1 provide consistent support for the primary hypothesis concerning beliefs about motivations for compliance with COVID-19 public health guidance. Across all the three measures, the standard emphasis on the desire to protect others relative to the obligation to the community was greater among the United States participants than among the Chinese participants.

## Study 2

In Study 1, we asked the participants to report their beliefs about the motivations of third parties for compliance with COVID-19 public health guidance. In Study 2, we added items asking the participants to report their own motivations for compliance. Since the results of Study 1 indicated an emphasis on different mandates in different countries (masks in China, social distancing in the United States), we included separate measures about motivations for compliance with each mandate.^2^

### Method

#### Participants

We conducted Study 2 at the end of May 2020. As in Study 1, the a priori power analysis indicated that a sample of 200 participants would provide 80% power to detect a small effect (f = 0.1) or larger of the interaction between country and motivation type. Therefore, we ended up recruiting 140 participants (51% women; age 18–90 with median of 56 years) residing in the United States from TurkPrime and 133 participants (52% women; age 18–49 with median of 28 years) residing in China from Sojump. Responses of the United States participants indicated that 80% self-identified as White, 5.7% Black, 5% Asian or Pacific Islander, 2.9% multi-ethnic, 2.1% Latino, and 0.7% American Indian/Alaskan Native. Responses of the Chinese participants indicated that 95.5% self-identified as Han.

#### Measures and Procedure

The participants completed an online survey. They first read two short vignettes in which the protagonist followed COVID-19 public health guidance regarding face masks (vignette 1) and social distancing (vignette 2). They then reported their beliefs about the motivations of the protagonist for compliance. We asked the participants to report their beliefs about the motivations of each protagonist *via* the *importance rating* measure we used in Study 1.

The participants then reported their own motivations for compliance with COVID-19 public health guidance *via* both the *relative importance (*percentage) measure and *importance rating* (scale) measure from Study 1. Since the countries were then at different stages of the COVID-19 pandemic—cases had decreased markedly in China but were still rising in the United States—we asked the participants to imagine their responses to the future waves of COVID-19 infections.

Finally, the participants responded to several items about their experience of the pandemic (e.g., “I have been diagnosed with coronavirus”; “I know someone in my social network who died because of Coronavirus”). They then completed demographic questions. Again, we developed all materials in English, translated into Chinese, and then back-translated into English to identify and correct any discrepancy.

### Results

Correlations among different motivations for the Unites States and Chinese participants appear in Tables 6, 7, respectively. As in Study 1, we first conducted mixed-model ANOVAs with motivation type (for which the number of levels varied according to the measure) as the within-participant variable and country (China and Unites States) as the between-subject variable. We then conducted mixed-model ANOVAs with motivation type (*desire to protect others* or *obligation to the community*) as the two-level within-participant variable and country as the between-subject variable.

**Table 6:** Correlations among different motivations—United States participants.

**Variable**	**1**	**2**	**3**	**4**	**5**	**6**	**7**	**8**	**9**	**10**	**11**	**12**	**13**
**Face mask (other)**													
1. Desire													
2. Obligation	0.807^*^												
3. Self-protection	0.577^**^	0.600^**^											
**Social distance (other)**													
4. Desire	0.592^**^	0.564^**^	0.487^**^										
5. Obligation	0.636^**^	0.563^**^	0.478^*^	0.845^**^									
6. Self-protection	0.403^**^	0.352^**^	0.542^**^	0.795^**^	0.722^**^								
**Relative importance (own)**													
7. Desire	−0.171^*^	−0.166	−0.303^**^	−0.237^**^	−0.256^**^	−0.288^**^							
8. Obligation	0.116	0.115	0.057	0.132	0.031	0.055	0.044						
9. Self–protection	0.043	0.040	0.177^*^	0.079	0.161	0.167^*^	−0.741^**^	−0.703^**^					
**Importance rating (own)**													
10. Desire	0.426^**^	0.429^**^	0.434^**^	0.499^**^	0.407^**^	0.401^**^	−0.112	0.053	0.044				
11. Obligation	0.441^**^	0.441^**^	0.329^**^	0.531^**^	0.447^**^	0.439^**^	−0.305^**^	0.201^*^	0.081	0.742^**^			
12. Self–protection	0.267^**^	0.283^**^	0.498^**^	0.268^**^	0.265^**^	0.405^**^	−0.445^**^	−0.037	0.341^**^	0.607^**^	0.527^**^		
13. Punishment	−0.050	0.058	0.037	0.143	0.111	0.121	−0.134	−0.079	0.148	−0.118	−0.035	−0.109	
14. Authority	0.106	0.141	0.256^**^	0.139	0.138	0.060	−0.148	0.096	0.041	0.206^*^	0.298^**^	0.159	0.455^**^

*Numbers indicate bivariate Pearson Correlation*.

* *p < 0.05*,

** *p < 0.01*.

**Table 7:** Correlations among different motivations—Chinese participants.

**Variable**	**1**	**2**	**3**	**4**	**5**	**6**	**7**	**8**	**9**	**10**	**11**	**12**	**13**
**Face mask (other)**													
1. Desire													
2. Obligation	0.405^**^												
3. Self-protection	0.103	0.170^*^											
**Social distance (other)**													
4. Desire	0.618^**^	0.303^**^	0.037										
5. Obligation	0.271^**^	0.532^**^	0.118	0.482^**^									
6. Self-protection	0.069	−0.092	0.563^**^	−0.026	0.099								
**Relative importance (own)**													
7. Desire	0.045	−0.065	−0.251^**^	0.140	−0.030	−0.146							
8. Obligation	0.065	0.037	−0.034	0.096	0.096	−0.131	−0.086						
9. Self–protection	−0.082	0.020	0.209^*^	−0.174^*^	−0.050	0.205^*^	−0.666^**^	−0.686^**^					
**Importance rating (own)**													
10. Desire	0.373^**^	0.290^**^	−0.032	0.378^**^	0.367^**^	0.062	0.256^**^	0.011	−0.195^*^				
11. Obligation	0.409^**^	0.389^**^	0.189^*^	0.296^*^	0.297^**^	0.118	−0.030	0.237^**^	−0.200^*^	0.416^**^			
12. Self–protection	0.087	0.214^**^	0.347^**^	0.030	0.175^*^	0.341^**^	−0.299^**^	−0.211^*^	0.376^**^	0.068	0.096		
13. Punishment	0.053	0.046	−0.155	−0.090	0.097	−0.093	0.145	−0.085	−0.042	0.199^*^	0.095	−0.212^*^	
14. Authority	0.151	0.176^*^	−0.004	−0.224^**^	0.138	−0.071	0.118	0.073	−0.141	0.174^*^	0.397^**^	−0.037	0.457^**^

*Numbers indicate bivariate Pearson Correlation*.

* *p < 0.05*,

** *p < 0.01*.

#### Motivation of Others

**Figure 4:**
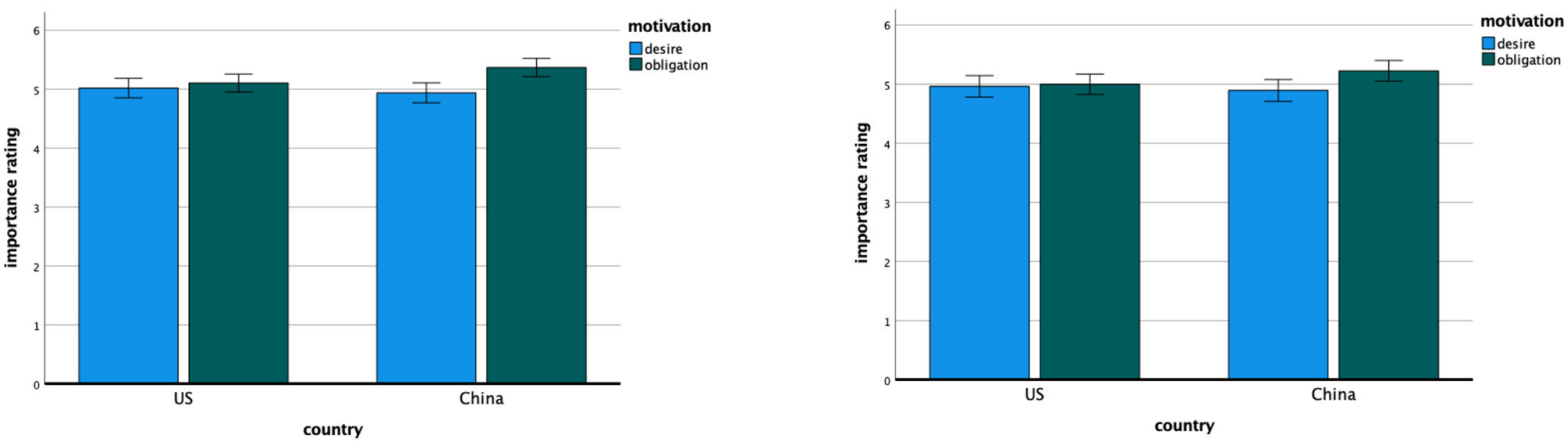
Study 2—the importance ratings of desire and obligation motivation for wearing face masks (left) and keeping social distance (right) for American and Chinese participants. Error bars denote 95% confidence intervals of the mean.

Means and standard deviations for *importance ratings* of different motivations for compliance of a protagonist with different mandates appear in Table 8. For the face mask mandate, the overall 2 (China, United States) × 3 (desire, obligation, self-protection) ANOVA revealed the anticipated interaction, *F*_(2, 542)_ = 12.18, *p* < 0.001, η^2^ = 0.043, indicating that the participants in Chinese and United States settings diverged in their relative emphasis on different motivations as an explanation for compliance of the protagonist with wearing face masks mandate. More importantly, the focused analysis revealed a significant main effect of motivation type, this time in the opposite direction as in Study 1—such that the participants indicated greater importance of *obligation to the community* (M = 5.23, SD = 0.92) than *desire to protect others* (M = 4.98, SD = 0.99), *F*_(1, 271)_ = 29.89, *p* < 0.001, η^2^ = 0.099—qualified by the hypothesized interaction with country, *F*_(1, 271)_ = 13.29, *p* < 0.001, η^2^ = 0.047. Consistent with the primary hypothesis (and as shown in Figure 4), the relative emphasis on *desire to protect others* over *obligation to the community* was greater (in this case, less negative) for the United States participants, *t*(139) = −1.51, *p* = 0.134, Cohen's *d*_*z*_ = −0.13, 95% CI [−0.29, 0.04] than for the Chinese participants, *t*(132) = −5.67, *p* < 0.001, Cohen's *d*_*z*_ = −0.49, 95% CI [−0.67, −0.31]. The overall country difference was not significant.

**Table 8:** Descriptive data for the belief of the protagonist's motivation—Chinese and American participants.

**Motivation**	**Face mask mandate**				***t***(271)****	**Cohen's *d_***s***_***	**Social distance mandate**				***t***(271)****	**Cohen's *d_***s***_***
	**US**		**China**				**US**		**China**			
	**M**	**SD**	**M**	**SD**			**M**	**SD**	**M**	**SD**		
Desire	5.02	1.08	4.94	0.89	0.68	0.082	4.96	1.16	4.89	1.02	0.53	0.064
Obligation	5.11	1.09	5.37	0.67	−2.37^*^	−0.287	5.00	1.25	5.23	0.75	−1.81	−0.219
Self–protection	5.25	1.08	5.69	0.54	−0.42^***^	−0.507	5.22	1.10	5.63	0.62	−3.70^***^	−0.448

* *p < 0.05*,

*** *p < 0.001*.

We found similar results for the social distancing mandate. The overall 2 (China, United States) × 3 (desire, obligation, self-protection) ANOVA revealed the anticipated interaction, *F*_(2, 540)_ = 9.68, *p* < 0.001, η^2^ = 0.035. More importantly, the focused analysis revealed a significant main effect of motivation type—again in the opposite direction as in Study 1, such that the participants indicated greater importance of *obligation to the community* (M = 5.11, SD = 1.04) than *desire to protect others* (M = 4.93, SD = 1.09), *F*_(1, 271)_ = 14.09, *p* < 0.001, η^2^ = 0.049—qualified by the hypothesized interaction with country, *F*_(1, 271)_ = 9.13, *p* = 0.003, η^2^ = 0.033. Consistent with the primary hypothesis (and as shown in Figure 4), the relative emphasis on *desire to protect* others over *obligation to the community* was greater (again, in this case, less negative) for the United States participants, *t*(139) = −0.63, *p* = 0.531, Cohen's *d*_*z*_ = −0.05, 95% CI [−0.22, 0.11] than for the Chinese participants, *t* (132) = −4.12, *p* < 0.001, Cohen's *d*_*z*_ = −0.36, 95% CI [−0.53, −0.18]. The overall country difference was not significant.

#### Own Motivation

**Figure 5:**
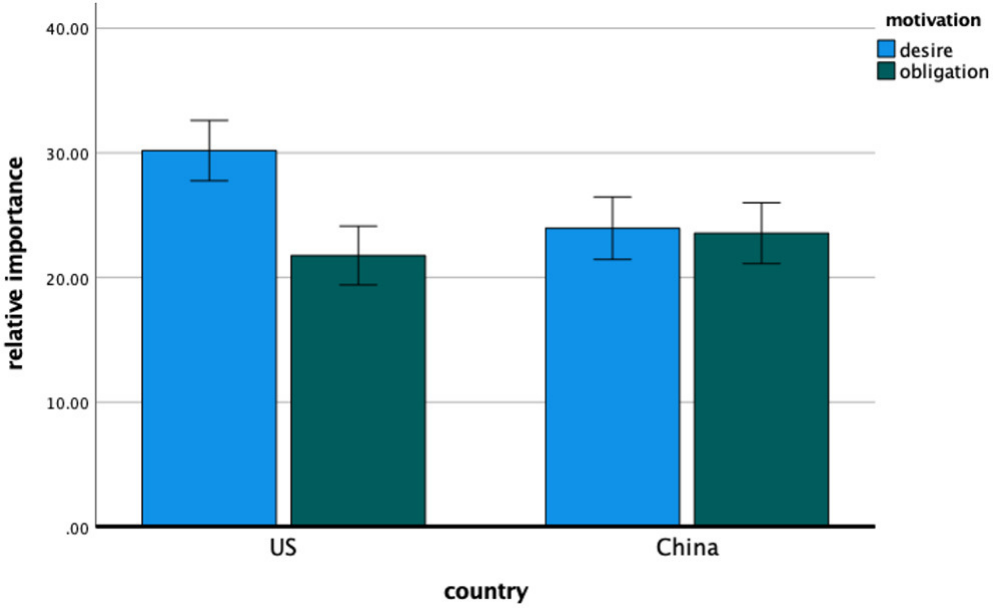
Study 2—the relative importance of desire and obligation motivation for the self for American and Chinese participants. Error bars denote 95% confidence intervals of the mean.

Means and standard deviations for belief of the participants about their own motivations appear in Table 9. For the *relative importance measure*, we again observed an overall 2 (China, United States) × 3 (desire, obligation, self-protection) interaction, *F*_(2, 546)_ = 5.07, *p* = 0.011, η^2^ = 0.018, indicating that the participants in Chinese and United States settings diverged in their relative emphasis on different motivations as an explanation for their own motivations for compliance with COVID-19 measures. More importantly, the focused analysis revealed a significant main effect of motivation type—such that, similar to Study 1, the participants reported greater importance of *desire to protect others* (M = 27.18, SD = 14.97) than *obligation to the community* (M = 22.64, SD = 14.27), *F*_(1, 273)_ = 12.69, *p* < 0.001, η^2^ = 0.044—qualified by the hypothesized interaction with country, *F*_(1, 273)_ = 10.5, *p* < 0.001, η^2^ = 0.037. Consistent with the primary hypothesis and results of Study 1 (and as shown in Figure 5), the relative emphasis on desire to protect others over obligation to the community as a motivation for compliance with COVID-19 guidance was greater for the United States participants, *t*(141) = 4.53, *p* < 0.001, Cohen's *d*_*z*_ = 0.38, 95% CI [0.21, 0.55] than for the Chinese participants, *t*(132) = 0.25, *p* = 0.805, Cohen's *d*_*z*_ = 0.02, 95% CI [−0.15, 0.19].

**Table 9:** Descriptive data for Chinese and American participants' own motivations.

**Motivation**	**Relative importance**				***t***(271)****	**Cohen's *d_***s***_***	**Importance rating**				***t***(271)****	**Cohen's *d_***s***_***
	**US**		**China**				**US**		**China**			
	**M**	**SD**	**M**	**SD**			**M**	**SD**	**M**	**SD**		
Desire	30.19	16.48	23.96	12.45	3.52^**^	0.425	5.07	0.95	4.78	0.85	2.65^**^	0.319
Obligation	21.77	15.56	23.56	12.76	−1.04	−0.125	4.84	1.03	5.27	0.75	−3.98^***^	−0.481
Self–protection	48.04	23.16	52.47	17.04	−1.80	−0.217	5.26	1.01	5.74	0.58	−4.76^***^	−0.575
Punishment							2.79	1.52	3.35	1.41	−3.15^**^	−0.381
Authority							3.73	1.52	4.11	1.45	−2.13^*^	−0.258

* *p < 0.05*,

** *p < 0.01*,

*** *p < 0.001*.

**Figure 6:**
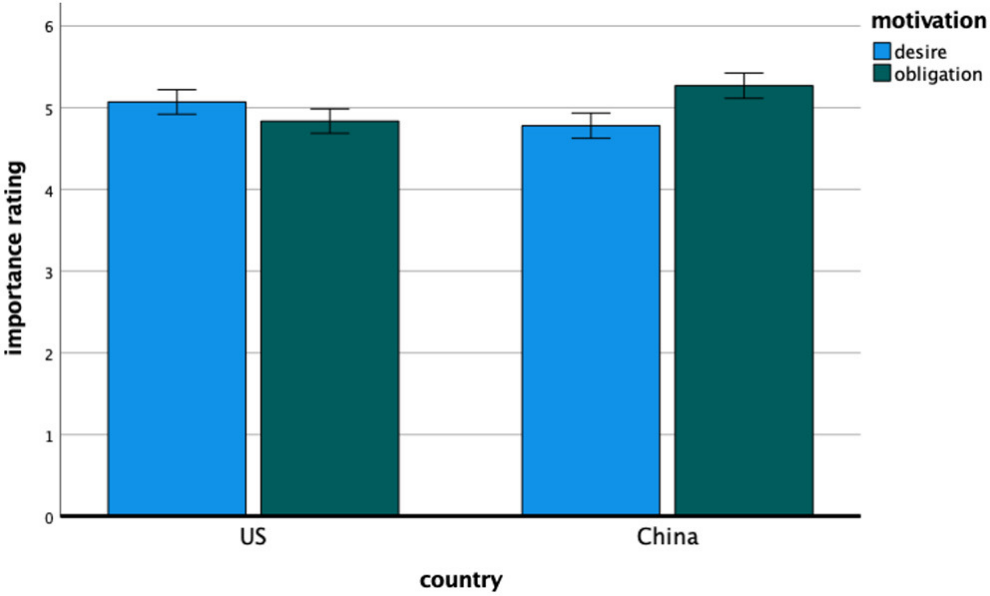
Study 2—the importance rating of desire and obligation motivation for the self for American and Chinese participants. Error bars denote 95% confidence intervals of the mean.

For the *importance ratings measure*, we also found an overall 2 (China, United States) × 5 (desire, obligation, self-protection, punishment, authority) interaction, *F*_(4, 1, 088)_ = 7.61, *p* < 0.001, η^2^ = 0.027. In this case, and unlike Study 1, the focused analysis revealed a significant main effect of motivation type, such that the participants reported greater importance of *obligation to community* (M = 5.05, SD = 0.93) than *desire to protect others* (M = 4.93, SD = 0.91), *F*_(1, 272)_ = 7.08, *p* = 0.008, η^2^ = 0.025. More importantly, the hypothesized interaction between motivation type and country qualified this main effect, *F*_(1, 272)_ = 57.03, *p* < 0.001, η^2^ = 0.173. Consistent with the primary hypothesis and results of Study 1 (and as shown in Figure 6), the emphasis on *desire to protect others* over *obligation to the community* as a motivation for their own compliance with COVID-19 guidance was greater for the United States participants, *t*(140) = 3.9, *p* < 0.001, Cohen's *d*_*z*_ = 0.33, 95% CI [0.16, 0.5], than it was for the Chinese participants, who instead showed a tendency to emphasize *obligation to the community* over *desire to protect others, t*(132) = −6.5, *p* < 0.001, Cohen's *d*_*z*_ = −0.56, 95% CI [−0.75, −0.38]. The overall country difference was not significant.

#### Control Variables

As in Study 1, we included responses to demographic items and experience of coronavirus as covariates and re-ran the analyses described above. The results of these analyses do not substantially qualify the conclusions that we reported above. Specifically, the *p*-value associated with the focal interaction remained statistically significant (i.e., *p* < 0.05) in all cases except one. The sole exception was the analysis for motivation of another to wear a face mask, for which the focal interaction was only marginally significant (*p* = 0.078). This again provides some assurance that observed results were not a function of demographic differences in the samples.

#### Summary

Results of Study 2 provide consistent support for the primary hypothesis concerning beliefs about own motivations and those of others for compliance with COVID-19 public health guidance. Across all the measures, the standard emphasis on the desire to protect others relative to the obligation to the community was greater among the United States participants than among the Chinese participants.

## Discussion

This study investigated a hypothesis regarding cultural variation in beliefs about prosocial motivations for compliance with COVID-19 public health guidance. In two studies, we found that the relative emphasis on the desire to protect others rather than the obligation to the community as an explanation for compliance was greater among the United States participants than the Chinese participants. We observed this pattern for explanations of both own motivations and those of others for compliance with both social distancing and face mask mandates.

The account of this pattern emphasizes the distinction between cultural-ecological affordances for ontological experience—in terms of embeddedness and interdependence vs. abstraction and independence—that are prominent in Chinese and American settings, respectively (Markus and Kitayama, 1991; Markus et al., 1997). In the cultural ecologies of abstracted independence that constitute everyday life in many United States settings, an ontological experience as a bounded entity abstracted from context is associated with an experience of the motivation for behavior in terms of personal desire and choices. In the cultural ecologies of embedded interdependence prominent in many Chinese settings, the ontological experience as a relational node embedded in context is associated with an experience of the motivation for behavior in terms of social expectations and obligation. Having observed hypothesized differences in the comparison of the participants from Chinese and United States settings, a desirable next step will be to determine whether these differences generalize to other cultural ecologies of embedded interdependence (e.g., African settings and rural areas) and abstracted independence (e.g., European countries and urban areas).

### Patterns Across Studies

Although the results across studies provided consistent support for the primary hypothesis, they also revealed interesting differences. For example, consistent with the idea of self-serving bias in attributions, the participants in both settings indicated greater relative importance of self-interest motivation in the case of others (Study 1, Table 3) than self (Study 2, Table 9). Similarly, they appeared to rate prosocial motivation as less important but (at least among the United States participants) self-interest as more important, as an explanation for compliance of others (Study 1, Table 4) than the compliance of self (Study 2, Table 9).

The other interesting pattern of variation across studies concerns a difference in important ratings of different prosocial motivations as an explanation for the behavior of others. Whereas, the Chinese participants consistently rated *obligation to the community* significantly more important than *desire to protect others*, patterns of ratings for the United States participants varied across the studies. Specifically, the United States participants rated *desire to protect others* significantly more important than *obligation to the community* as an explanation for compliance of self and others with general public health guidelines in Study 1 (Tables 4, 9 and Figures 2, 6), but they rated *obligation to the community* slightly but not significantly more important than *desire to protect others* as an explanation for compliance of others with mandates concerning both face masks and social distancing in Study 2 (Table 8 and Figure 4). Whether this pattern reflects differences in language used to describe the target of compliance (i.e., *guidelines* vs. *mandate*), increased importance of obligation as the pandemic progressed over time, or simply a form of random variation remains a question for further study.

The third interesting pattern concerns correlations between importance ratings for different motivations. Contrary to the secondary hypothesis (footnote 1), we did not observe stronger associations between ratings for *obligation to the community* and *desire to protect others* for the Chinese compared with the United States participants. Instead, we observed different patterns of relationship between ratings for *obligation to the c*ommunity and the other motivations across settings. The United States participants appeared to respond to all motivational sources in an undifferentiated, categorical fashion, such that importance ratings for *obligation to the community* were significantly associated with ratings for all other motivations (*desire to protect others, self-protection, concern for punishment*, and *obedience to authority*) except for *concern for punishment* in Study 2 (Tables 1, 6). In contrast, importance ratings for *obligation to the community* among the Chinese participants were more narrowly associated only with ratings for *desire to protect others* and not with ratings for *self-protection, concern for punishment*, and (except for Study 2) *obedience to authority* (Tables 2, 7). Whether this pattern reflects the compatibility of desire and obligation in the context of COVID-19 or generally stronger motivation to comply with public health guidance among the United States participants remains a matter for further investigation.

### Limitations and Future Directions

One limitation of these studies concerns demographic differences between the Chinese and the United States samples. Although we recruited samples in both settings from a large online subject pool, the Chinese samples were younger than the United States samples. Accordingly, it remains possible that observed variation in motivations between two countries is a product of this demographic difference, which is irrelevant to the theoretical interest of the authors in cultural ecologies of abstracted independence and embedded interdependence. To some extent, we ruled out this alternative explanation by showing that the observed results remained significant after including demographics as covariates. However, future studies with larger, more representative, and similar-aged samples can provide more conclusive results.

Another relevant limitation concerns differences in the trajectory of the pandemic across the two countries. At the time we conducted the studies, China had mostly recovered from the impact of COVID-19, was reporting very few new cases, and had relaxed many public health restrictions. In contrast, the United States was still reporting steady increases in new cases and heavily emphasized the importance of COVID-19 public health measures. One might speculate that motivations for compliance might vary at different phases of the COVID-19 pandemic. For example, obligation motivations may play a greater or lesser role when the threat of infection is high. To address this issue, we asked the participants in Study 2 to anticipate their motivations for compliance with measures in the future waves of COVID-19 infections. Although the results again revealed hypothesized patterns, a more definitive test of hypotheses awaits an opportunity to investigate explanations for compliance across settings at similar phases of the COVID-19 pandemic.

Finally, perhaps the most important limitation of these studies is that we examined belief about own motivations and those of others for compliance with COVID-19 measures. We did not attempt to assess actual motivations for compliance. If beliefs about the importance of different motivations reflect reality, then these results suggest an interesting hypothesis that actual motivations for compliance vary across settings. Accordingly, one might anticipate that activation of desire (compared with obligation) motivation would promote compliance with COVID-19 measures more effectively in United States settings than Chinese settings. The important implication is that attempts to motivate compliance with public health guidance by emphasizing the desire to protect others may be relatively more effective than appeals to the obligation in United States settings, but the reverse may be true in Chinese settings. This constitutes an important direction for future research with profound practical implications.

### Conclusions

A core contribution of a cultural psychology analysis is to denaturalize the WEIRD ways of being that dominant forms of psychological science tend to interpret as just-natural features of the human organism (e.g., Adams and Kurtiş, 2018). This study applies this perspective to the topic of prosocial motivation. Dominant or hegemonic perspectives of psychological science tend to portray the desire to protect others as a superior expression of prosocial motivation that explains compliance with public health guidance better than social obligation. In contrast, the results of this study are consistent with the proposition that the dominant valorization of personal desire and relative denigration of social obligation is not a reflection of natural human tendencies but instead reflects the basis of psychological science in WEIRD cultural ecologies that promote an experience of independence and abstraction from context.

By denaturalizing the WEIRD patterns that dominant perspectives portray as natural, a cultural psychology perspective helps to problematize those supposedly natural patterns, which prompts consideration of drawbacks that often remain obscure in dominant accounts. With respect to compliance with public health guidance during the COVID-19 pandemic, the current studies suggest an explanation for the association between the spread or severity of COVID-19 and cultural-ecological forces associated with the experience of abstracted independence (e.g., relational mobility) (Salvador et al., 2020; see also Webster et al., 2021). In particular, the experience of abstracted independence may inhibit the development of obligation-based motivation that is important to mobilize support for public health mandates. A conclusive test of this idea awaits future research.

More generally, a cultural psychology perspective on the COVID-19 pandemic helps to illuminate the public health costs from the neoliberal individualist lifeways characteristic of WEIRD modernity (Adams et al., 2019). These lifeways afford an experience of radical abstraction from context and a sense of personal freedom not only to pursue authentic desires and relations of one's choosing but also to disinvest in obligations, social formations, or even bodies of knowledge that one finds burdensome, undesirable, or inconvenient. Decolonial perspectives of cultural psychology suggest that these neoliberal individualist lifeways are not the innocent product of cultural development divorced from the political economy but instead reflect the *coloniality*—that is, enduring effects of colonialism on habits of mind and being (Maldonado-Torres, 2007)—inherent in the Eurocentric modern order (Richardson, 2019). Indeed, an influential account suggests that a primary motivation for neoliberal individualist disinvestment from notions of community, a civic obligation, and authoritative knowledge—particularly in the United States settings—may be a defense of racialized privilege and White supremacy (Metzl, 2019). Simply put, responses to the COVID-19 pandemic suggest the extent to which neoliberal individualist lifeways constitute a public health emergency.

## Data Availability Statement

The datasets presented in this study can be found in online repositories. The names of the repository/repositories and accession number(s) can be found below: https://osf.io/y34x8/.

## Ethics Statement

The studies involving human participants were reviewed and approved by University of Kansas. The ethics committee waived the requirement of written informed consent for participation.

## Author Contributions

TA contributed to the study design, data collection, data analyses and interpretation, and writing of the manuscript. GA contributed to the study design, data analyses and interpretation, and writing of the manuscript. XZ contributed to the study design, data interpretation, and revision of the manuscript. All the authors contributed to the article and approved the submitted version.

## Conflict of Interest

The authors declare that the research was conducted in the absence of any commercial or financial relationships that could be construed as a potential conflict of interest.

## Footnotes

^1^We pre-registered the hypotheses and methods https://aspredicted.org/blind.php?x=vb6e5e. In addition to the primary hypothesis, we also pre-registered and tested a secondary hypothesis, based on previous research (Buchtel et al., 2018), that the association between obligation and desire motivation is higher for the Chinese participants than the United States participants. Results across all three pairs of motivation measures provided no support for this secondary hypothesis, so we do not discuss it further. Details of these analyses appear in the Supplementary Material.

^2^We pre-registered our hypotheses and methods https://aspredicted.org/blind.php?x=2b5m9f. In addition to the primary hypothesis, we also pre-registered and tested a secondary hypothesis that people's belief of a third party's obligation motivation is positively correlated with the positive evaluation of the third party, and this correlation is stronger among Chinese participants. Results provided no support for this secondary hypothesis, so we do not discuss it further. Details of these analyses appear in the Supplementary Material.
