# Supplementary material for: Obligation or Desire: Variation in Motivation for Compliance With COVID-19 Public Health Guidance
Source: Front Psychol. 2021 Jul 6;12:647830. doi: 10.3389/fpsyg.2021.647830 (PMC8290058; doi:10.3389/fpsyg.2021.647830)
Supplement: Supplementary file 1 [file Data_Sheet_1.docx]

Supplementary Material

**Research Materials – Study 1**

**Relative importance measure**

In this study, we will ask you to explain people's behaviors. To explain what we want you to do, we will first show you an example. This example is about corn.

A model for explaining why the price of corn increased might include three factors:

(1) the state of the economy (e.g., is there a recession?).

(2) the availability of other grains (e.g., is this a good season for wheat?).

(3) corn yield that season (e.g., did much or little corn grow this season?).

Let’s imagine we think that the economy is the most important factor, that corn yield of is a close second, and that the availability of other grains is a distant third. If we had 100% to allocate to the different contributors, we might represent it as follows:

(1) State of the Economy (55%)

(2) Availability of other grains (5%)

(3) Corn production yield (40%)

In this study, you will do a similar task. Instead of the price of corn, we will ask you to consider why **people around you** are compliant with COVID-19 guidance.

Governments and public health officials have asked people to avoid large gatherings, to wear masks in public, to stay at home as much as possible, and other measures in response to the COVID-19 pandemic. Your task is to allocate 100 percentage points to indicate the relative importance of each of the following motivations as an explanation for why **people around you** are compliant with COVID-19 guidance.

1. **Desire for self-protection:** People want to protect themselves from COVID-19 disease.
2. **Desire to protect others:** People feel empathy or care for others and desire to protect them from the disease
3. **Obligation to the community**: As a member of the community, people feel a social responsibility to do their part in the community effort to stop the disease.

Note: the sum of (a), (b), and (c) 100%.

**Importance ratings measure**

Why are **people around you** compliant with COVID-19 guidance? Please use a scale from 0 (not at all) to 5 (very much) to rate the extent to which you think people are compliant for the following reasons.

People around me are compliant because of:

1. Concern for punishment
2. Obedience to authority
3. Obligation to the community
4. Desire to protect others
5. Desire for self-protection

**Motivation to Help Scale**

Authorities have urged people to wear face masks or other coverings in public spaces as a response to the COVID-19 pandemic. Alex has been following this guidance, wearing face masks to protect other people. What do you think is Alex’s motivation for doing so?

Alex has been wearing face masks to protect others:

1. So that he would be liked (C)

2. Because he’d feel like a bad person if he didn’t (C)

3. Because others would get mad at him if he didn’t (C)

4. Because he thought it was important to act in this way (A)

5. Because he liked acting this way (A)

6. Because he felt he had to (C)

7. Because he felt he should (C)

8. Because he valued doing so (A)

9. Because he cared about others in the community (A)

10. Because he thought he would enjoy it (A)

11. Because he appreciated that his help could be useful (A)

**COVID-19 questions**

Where do you stay since the COVID-19 outbreak? _____ (country & state)

Approximately, how many confirmed cases of COVID-19 are there in your state? _______

Are you living in an area with a “social distancing” mandate? Yes/No

Are you living in an area with a “wearing face mask” mandate? Yes/No

**Demographics**

Age

Please select your gender (If no option applies, please respond in comment box).

- Male
- Female
- Transgender Woman
- Transgender Man
- There is no option that applies to me

What is your family's income level each year?

- Less than $10,000
- $10,001 to $15,000
- $15,001 to $25,000
- $25,001 to $50,000
- $50,001 to $75,000
- $75,001 to $100,000
- more than $100,000
- Do not know

What is the highest level of education you completed?

- Attended high school
- Completed high school
- Attend or attended college
- Completed a university degree
- Attend or attended graduate school
- Completed a graduate degree
- Other

Please select the response that best describes your racial/ethnic background.

- White
- Black or African American
- American Indian or Alaska Native
- Asian
- Latina/o, Hispanic
- Native Hawaiian or Other Pacific Islander
- Multiracial (specify below)
- Other (please specify in the comment box)

How religious are you?

1 (Not Religious) 2 3 4 5 6 7 (Very Religious)

**Research Materials – Study 2**

**Wearing face mask**

The Public Health Officials urged people to **wear face masks or other coverings** in public spaces as a response to the COVID-19 pandemic. Alex followed this guidance during the time of the COVID-19 pandemic. S/He wore a face mask every time s/he went outside.

***Motivation***

Why did Alex wear a face mask every time he went outside? What do you think Alex’s **motivation** is for doing so? Please use a scale from 1 (strongly disagree) to 6 (strongly agree) to rate the extent to which you think Alex has been wearing a face mask for the following reasons.

Alex has been wearing face masks because of:

1. Obligation to the community: As a member of the community, Alex feels a social responsibility to do his/her part in the community effort to stop the disease.
2. Desire to protect others: Alex feels empathy or cares for others and desires to protect them from the disease
3. Desire for self-protection: Alex wants to protect himself/herself from COVID-19 disease.

***Evaluation***

Please rate the following items to indicate how you feel about Alex:

1 = strongly disagree; 6 = strongly agree

1. Alex is a **kind** person. (Warmth)
2. Alex is a **competent** person. (Competence)
3. Alex is a **caring** person. (Warmth)
4. Alex is a **responsible** person. (Competence)
5. Alex is a **warmhearted** person. (Warmth)
6. Alex is a **dependable** person. (Competence)

**Keeping social distance**

The Public health officials urged people to **keep social distance from others** in public spaces as a response to the COVID-19 pandemic. Robin followed this guidance during the time of the COVID-19 pandemic. S/He kept distance between him/herself and other people every time s/he went outside.

***Motivation***

Why did Robin keep social distance from others every time he went outside? What do you think Robin’s **motivation** is for doing so? Please use a scale from 0 (strongly disagree) to 6 (strongly agree) to rate the extent to which you think Robin has been keeping social distance for the following reasons.

Robin has been keeping social distance because of:

1. Obligation to the community: As a member of the community, Robin feels a social responsibility to do his/her part in the community effort to stop the disease.
2. Desire to protect others: Robin feels empathy or cares for others and desires to protect them from the disease
3. Desire for self-protection: Robin wants to protect himself/herself from COVID-19 disease.

**Evaluation**

Please rate the following items to indicate how you feel about Robin:

1 = strongly disagree; 6 = strongly agree

1. Robin is a **kind** person. (Warmth)
2. Robin is a **competent** person. (Competence)
3. Robin is a **caring** person. (Warmth)
4. Robin is a **responsible** person. (Competence)
5. Robin is a **warmhearted** person. (Warmth)
6. Robin is a **dependable** person. (Competence)

**Own motivation**

Governments and public health officials expect that recurring waves of COVID-19 infections will continue to affect societies that have survived the first wave of COVID-19 infections. These future waves once again will require governments and public health officials to ask people to avoid large gatherings, to wear masks in public, to stay at home as much as possible, and other measures in response to the COVID-19 pandemic.

***Relative importance measure***

We would like to know your motivations behind compliance behaviors. To explain what we want you to do, we will first show you an example. This example is about corn.

A model for explaining why the price of corn increased might include three factors:

(1) the state of the economy (e.g., is there a recession?).

(2) the availability of other grains (e.g., is this a good season for wheat?).

(3) corn yield that season (e.g., did much or little corn grow this season?).

Let’s imagine we think that the economy is the most important factor, that corn yield of is a close second, and that the availability of other grains is a distant third. If we had 100% to allocate to the different contributors, we might represent it as follows:

(1) State of the Economy (55%)

(2) Availability of other grains (5%)

(3) Corn production yield (40%)

You will do a similar task. Instead of the price of corn, we will ask you to consider why **you** would comply **with guidance about face masks, social distancing, and other measures in response to future waves of the COVID-19 pandemic.**

Your task is to allocate 100 percentage points to indicate how important you feel about each of the following motivations as an explanation for why **you** would comply with guidance about face masks, social distancing, and other measures in response to future waves of the COVID-19 pandemic.

1. **Desire for self-protection:** I want to protect myself from COVID-19 disease.
2. **Desire to protect others:** I feel empathy or care for others and desire to protect them from the disease
3. **Obligation to the community**: As a member of the community, I have a social responsibility to do their part in the community effort to stop the disease.

Note: the sum of (a), (b), and (c) 100%.

**Importance ratings measure**

Why would you comply with COVID-19 guidance? Please use a scale from 1 (strongly disagree) to 6 (strongly agree) to rate the extent to which you think you would comply for each of the following reasons.

I would comply because of:

1. Concern for punishment
2. Obedience to authority
3. Obligation to the community
4. Desire to protect others
5. Desire for self-protection

**COVID-19 questions**

- I have been diagnosed with coronavirus (COVID-19).
- I know someone in my social network who has been diagnosed with Coronavirus (COVID-19).
- I know someone in my social network who died because of Coronavirus (COVID-19).

**Demographics – same as in Study 1**

**Supplementary analyses – Study 1**

Table 1.

*Regression coefficients for obligation motivation as a function of desire motivation and country for three motivation measures.*

| Predicting variables | Relative importance | Importance rating | Motivation to help scale |
| --- | --- | --- | --- |
| Intercept | 11.34 | 3.81 | 3.52 |
| Desire | .28 | .89** | .49* |
| Country | 4.98* | .44** | .47** |
| Desire * Country | -.11 | -.20 | -.03 |
| *R^2^* | .054 | .38 | .34 |

Note: All coefficients represent unstandardized regression coefficients. * *p* < .05. ** *p* < .001. n = 226.

We tested a secondary hypothesis that the association between obligation and desire motivation is higher for Chinese participants than U.S. participants. We ran three regressions (one for each measure), predicting obligation motivation from desire motivation, country, and the interaction between desire motivation and country. Results showed that desire motivation positively predicted obligation for both Importance rating measure and Motivation to Help scale. This indicated the higher desire participants reported, the higher obligation motivation they would also report. However, desire motivation did not predict obligation for Relative importance measure, potentially due to its zero-sum character (different motivations are likely to be negatively correlated). More importantly and relevant to the hypothesis, the interaction between country and desire motivation did not predict obligation across three measures. This indicated that there was no significant difference in the association between Chinese (Relative importance, *β* = .06, *p* = .518; Importance rating, *β* = .50, *p* < .001; Motivation to help scale, *β* = .37, *p* < .001) and US participants (Relative importance, *β* = .21, *p* = .031; Importance rating, *β* = .66, *p* < .001; Motivation to help scale, *β* = .49, *p* < .001). See Table 1 and Table 2 in the manuscript for the correlations between obligation and desire motivations.

Table 2.

*Correlations between demographic variables and different motivations in three measures – U.S. participants.*

| Variable | distance | mask | gender | age | | income | Subjective SES |
| --- | --- | --- | --- | --- | --- | --- | --- |
| Distance |  |  |  |  | |  |  |
| Mask | -.006 |  |  |  | |  |  |
| Gender | -.019 | -.135 |  |  | |  |  |
| Age | .030 | -.176 | .213* |  | |  |  |
| Income | -.015 | .213* | -.181 | -.065 | |  |  |
| Subjective SES | .058 | .254** | -.219* | .083 | | .375** |  |
| Relative importance | | | | |  |  |  |
| 1. Desire | .002 | .069 | -.009 | -.171 | | .122 | .116 |
| 2. Obligation | .001 | -.076 | -.071 | -.079 | | -.069 | .180 |
| 3. Self-protection | -.002 | -.004 | .048 | .166 | | -.045 | -.185 |
| Importance rating | | | | |  |  |  |
| 4. Desire | -.066 | .123 | .000 | .050 | | -.012 | .050 |
| 5. Obligation | -.058 | .094 | -.074 | .033 | | .039 | .086 |
| 6. Self-protection | .017 | -.006 | .071 | .252* | | -.126 | -.025 |
| 7. Punishment | .079 | .258** | -.295** | -.397** | | .174 | .319** |
| 8. Authority | .024 | .021 | -.076 | -.060 | | .021 | .165 |
| Motivation to Help | | | | |  |  |  |
| 9. Autonomous | .012 | .094 | -.005 | .020 | | .040 | .094 |
| 10. Controlled | -.028 | .186 | -.127 | -.054 | | .054 | .209* |

Numbers indicate bivariate Pearson Correlation.

**p < .05. **p < .01.*

Table 3.

*Correlations between demographic variables and different motivations in three measures – Chinese participants.*

| Variable | distance | mask | gender | age | | income | Subjective SES |
| --- | --- | --- | --- | --- | --- | --- | --- |
| Distance |  |  |  |  | |  |  |
| Mask | .160 |  |  |  | |  |  |
| Gender | -.087 | -.008 |  |  | |  |  |
| Age | -.001 | .104 | -.106 |  | |  |  |
| Income | -.069 | .080 | -.014 | .239* | |  |  |
| Subjective SES | -.115 | -.090 | .003 | .145 | | .314** |  |
| Relative importance | | | | |  |  |  |
| 1. Desire | .093 | .074 | -.052 | -.031 | | .030 | .103 |
| 2. Obligation | .029 | -.029 | -.073 | -.057 | | .006 | .042 |
| 3. Self-protection | -.082 | -.029 | .086 | .061 | | -.024 | -.099 |
| Importance rating | | | | |  |  |  |
| 4. Desire | -.027 | -.034 | -.068 | -.025 | | .109 | .110 |
| 5. Obligation | -.094 | -.060 | -.065 | -.016 | | .079 | .095 |
| 6. Self-protection | .024 | -.065 | .120 | .081 | | .057 | -.004 |
| 7. Punishment | .085 | -.074 | .008 | .051 | | .015 | .070 |
| 8. Authority | -.044 | -.078 | -.015 | .032 | | .110 | .172 |
| Motivation to Help | | | | |  |  |  |
| 9. Autonomous | .051 | .008 | .033 | .061 | | -.007 | .137 |
| 10. Controlled | .008 | .022 | -.067 | -.052 | | -.007 | .128 |

Numbers indicate bivariate Pearson Correlation.

**p < .05. **p < .01.*

**Analyses that include demographic variables as covariates**

### We conducted mixed-model ANOVAs with motivation type (desire to protect others and obligation to the community) as the within-participant variable, country (China and U.S.) as the between-subject variable, and gender, age, yearly income, subjective socioeconomic status, and the enforcement of face mask and social distance mandates as covariates.

### Relative importance

Our analysis revealed a significant interaction effect between motivation type and country, *F* (1, 205) = 10.93, *p* < .001, *η^2^* = .051. The main effect of motivation type, or the interaction between motivation type and demographic variables were not significant.

### Importance ratings

Our analysis revealed a significant interaction effect between motivation type and country, *F* (1, 204) = 5.735, *p* = .018, *η^2^* = .027. The main effect of motivation type, or the interaction between motivation type and demographic variables were not significant.

### Motivation to help

The interaction effect between motivation type and country was not significant, *F* (1, 205) = .237, *p* = .627, *η^2^* = .001. The interaction between motivation type and gender was marginally significant, *F* (1, 205) = 2,89, *p* = .091, *η^2^* = .014. The main effect of motivation type, or the interaction between motivation type and other demographic variables were not significant.

**Supplementary analyses – Study 2**

Table 4.

*Regression coefficients for obligation motivation as a function of desire motivation and country for three motivation measures.*

| Predicting variables | Face mask mandate | Social distance mandate | Relative importance-self | Importance rating-self |
| --- | --- | --- | --- | --- |
| Intercept | 5.07 | 4.97 | 21.65 | 4.72 |
| Desire | .82** | .91** | .04 | .80** |
| Country | .31** | .27** | 1.65 | .60** |
| Desire * Country | -.52** | -.56** | -.13 | -.43** |
| *R^2^* | .53 | .60 | .008 | .46 |

Note: All coefficients represent unstandardized regression coefficients. ** *p* < .001. n = 226.

We tested a secondary hypothesis that the association between obligation and desire motivation is higher for Chinese participants than U.S. participants. We ran four regressions (one for each measure), predicting obligation motivation from desire motivation, country, and the interaction between desire motivation and country. Results showed that desire motivation positively predicted obligation for Importance rating measures (Face mask mandate, Social distance mandate, and Importance rating-self). This indicated the higher desire participants reported, the higher obligation motivation they would also report. More importantly and relevant to the hypothesis, the interaction between country and desire motivation significantly predicted obligation for three Importance rating measures. This indicated that there were significant differences in the association between obligation and desire motivation between two countries. After probing the interaction, we found that contrary to the hypothesis, the associations were larger among US participants (Face mask mandate, *β* = .81, *p* < .001; Social distance mandate, *β* = .85, *p* < .001; and Importance rating-self, *β* = .74, *p* < .001) than Chinese participants (Face mask mandate, *β* = .41, *p* < .001; Social distance mandate, *β* = .48, *p* < .001; and Importance rating-self, *β* = .42, *p* < .001). None of the effect reported above were significant for Relative importance-self measure, potentially due to its zero-sum character. See Table 6 and Table 7 in the manuscript for the correlations between obligation and desire motivations.

Table 5.

*Regression coefficients for competence evaluation as a function of obligation motivation and country for face mask and social distance mandates.*

| Predicting variables | Face mask | Social distance |
| --- | --- | --- |
| Intercept | 3.26 | 3.31 |
| Obligation | .38** | .38** |
| Country | .07 | -.62 |
| Obligation * Country | -.08 | .05 |
| *R^2^* | .22 | .32 |

Note: All coefficients represent unstandardized regression coefficients. * *p* < .05. ** *p* < .001. n = 273.

We tested a secondary hypothesis that people’s belief of a third party’s obligation motivation is positively correlated with the positive evaluation of the third party, and this correlation is stronger among Chinese participants. We ran two regressions (one for face mask and the other for social distance mandate), predicting the evaluation of the third party’s competence from people’s belief of the third party’s obligation motivation, country, and the interaction between obligation and country. Results showed that for both mandates, obligation motivation positively predicted higher evaluation of competence. This indicated the higher obligation motivation people believed the third party had, the higher competence people would evaluate that third party. However, the interaction between obligation and country was not significant. This indicated the there was no difference in the association between obligation motivation and competence evaluation between Chinese (face mask, *β* = .30, *p* < .001; social distance, *β* = .43, *p* < .001) and US participants (face mask, *β* = .38, *p* < .001; social distance, *β* = .38, *p* < .001).

Table 6.

*Correlations between demographic variables and different motivations in three measures – U.S. participants.*

| Variable | Self-diagnosis | Other diagnosis | gender | age | | income | Subjective SES |
| --- | --- | --- | --- | --- | --- | --- | --- |
| Self-diagnosis |  |  |  |  | |  |  |
| Other diagnosis | .296** |  |  |  | |  |  |
| Gender | -.137 | -.036 |  |  | |  |  |
| Age | .070 | .029 | -.172* |  | |  |  |
| Income | -.190* | -.111 | .043 | -.034 | |  |  |
| Subjective SES | -.138 | -.013 | .144 | .087 | | .456** |  |
| Face mask (other) | | | | |  |  |  |
| 1. Obligation | .015 | .115 | -.188* | .241** | | .043 | .001 |
| 2. Desire | .003 | .179* | -.191* | .247** | | .066 | -.035 |
| 3. Self-protection | -.056 | .102 | -.145 | .106 | | -.089 | -.091 |
| Social distance (other) | | | | |  |  |  |
| 4. Obligation | .000 | .103 | -.117 | .135 | | -.028 | -.062 |
| 5. Desire | .038 | -.105 | -.109 | .151 | | -.037 | -.060 |
| 6. Self-protection | .031 | -.042 | -.030 | .090 | | -.037 | -.047 |
| Relative importance (Own) | | | | |  |  |  |
| 7. Self-protection | -.016 | .054 | -.012 | .093 | | -.034 | -.066 |
| 8. Desire | -.041 | -.075 | .017 | -.095 | | .155 | .073 |
| 9. Obligation | .067 | -.001 | .001 | -.042 | | -.114 | .021 |
| Importance rating (Own) | | | | | | | |
| 10. Punishment | -.149 | -.032 | .029 | -.249** | | -.065 | .124 |
| 11. Obedient | -.059 | .029 | -.131 | .007 | | -.223** | -.103 |
| 12. Obligation | .169* | .042 | -.149 | .171* | | -.062 | -.047 |
| 13. Desire | .166* | .037 | -.179* | .159 | | .021 | -.095 |
| 14. Self-protection | -.010 | -.083 | -.097 | .257** | | -.114 | -.138 |

Numbers indicate bivariate Pearson Correlation.

**p < .05. **p < .01.*

Table 7.

*Correlations between demographic variables and different motivations in three measures – Chinese participants.*

| Variable | Self-diagnosis | Other diagnosis | gender | age | | income | Subjective SES |
| --- | --- | --- | --- | --- | --- | --- | --- |
| Self-diagnosis |  |  |  |  | |  |  |
| Other diagnosis | .211* |  |  |  | |  |  |
| Gender | -.005 | -.091 |  |  | |  |  |
| Age | -.064 | -.021 | .156 |  | |  |  |
| Income | .113 | -.048 | .180* | -.269** | |  |  |
| Subjective SES | .081 | -.157 | .116 | .211* | | .461** |  |
| Face mask (other) | | | | |  |  |  |
| 1. Obligation | .254** | .014 | -.013 | .051 | | .065 | .002 |
| 2. Desire | .061 | -.020 | -.036 | .085 | | .160 | .150 |
| 3. Self-protection | .274** | -.048 | -.036 | -.036 | | .214* | .144 |
| Social distance (other) | | | | |  |  |  |
| 4. Obligation | .204* | .062 | .133 | -.059 | | .031 | -.027 |
| 5. Desire | .048 | .041 | .159 | -.043 | | .103 | .073 |
| 6. Self-protection | .026 | .013 | -.035 | .012 | | .139 | .117 |
| Relative importance (Own) | | | | |  |  |  |
| 7. Self-protection | .153 | .034 | -.060 | -.099 | | -.024 | -.125 |
| 8. Desire | -.115 | -.014 | .060 | .171 | | -.097 | .057 |
| 9. Obligation | -.092 | -.031 | .021 | -.037 | | .127 | .111 |
| Importance rating (Own) | | | | | | | |
| 10. Punishment | -.101 | -.283** | -.039 | -.181* | | -.234* | -.164 |
| 11. Obedient | -.076 | -.086 | -.065 | -.029 | | -.028 | .032 |
| 12. Obligation | .127 | .005 | -.007 | -.017 | | .116 | -.027 |
| 13. Desire | .115 | -.090 | .178* | .148 | | .177* | .033 |
| 14. Self-protection | .266** | .062 | -.083 | -.024 | | .135 | -.046 |

Numbers indicate bivariate Pearson Correlation.

**p < .05. **p < .01.*

**Analyses that include demographic variables as covariates**

### We conducted mixed-model ANOVAs with motivation type (desire to protect others and obligation to the community) as the within-participant variable, country (China and U.S.) as the between-subject variable, and gender, age, yearly income, subjective socioeconomic status, and whether the self and close others have been diagnosed with COVID as covariates.

### Other motivation (face mask)

Our analysis revealed a marginally significant interaction effect between motivation type and country, *F* (1, 250) = 3.13, *p* = .078, *η^2^* = .012. The main effect of motivation type, or the interaction between motivation type and demographic variables were not significant.

### Other motivation (social distance)

Our analysis revealed a significant interaction effect between motivation type and country, *F* (1, 250) = 4.20, *p* = .041, *η^2^* = .017. The main effect of motivation type, or the interaction between motivation type and demographic variables were not significant.

**Own motivation (relative importance measure)**

Our analysis revealed a significant interaction effect between motivation type and country, *F* (1, 251) = 4.22, *p* = .041, *η^2^* = .017. The main effect of motivation type, or the interaction between motivation type and demographic variables were not significant.

**Own motivation (importance ratings measure)**

Our analysis revealed a significant interaction effect between motivation type and country, *F* (1, 251) = 27.69, *p* < .001, *η^2^* = .099. The interaction between motivation type and gender was marginally significant, *F* (1, 251) = 3.31, *p* = .070, *η^2^* = .013. The main effect of motivation type, or the interaction between motivation type and other demographic variables were not significant.
